# A pilot study comparing the rehabilitation functional outcomes of post-COVID-19 stroke and non-COVID stroke patients: An occupational therapy perspective

**DOI:** 10.5339/qmj.2024.70

**Published:** 2024-12-31

**Authors:** Thajus Asirvatham, Reetha Sukumaran, Premraj Issac Chandran, Ajay Boppana, Mohammed Nasser Awadh

**Affiliations:** ^1^Hamad Medical Corporation, Doha, Qatar *Correspondence: Thajus Asirvatham. Email: TAsirvatham@hamad.qa

**Keywords:** Functional gain, stroke, post-COVID-19, hand function, cognition, occupational therapy

## Abstract

**Background and purpose:** Recent studies have highlighted the clinical characteristics and incidence of post-COVID-19 stroke conditions. Comparing the function and overall prognosis of stroke patients and post-COVID-19 stroke patients is an intriguing idea. Therefore, the aim of this study was to examine and compare the functional outcomes between the two groups from an occupational therapy perspective.

**Methods:** Forty patients admitted to a rehabilitation facility were included, 20 of whom were diagnosed with post-COVID-19 stroke and 20 with non-COVID-19 stroke (ischemic and hemorrhagic). The study was a mixed design consisting of both prospective and retrospective data collection. Existing data from electronic medical records were used for the retrospective dataset. The retrospective dataset only consisted of data from post-COVID-19 stroke patients. The prospective dataset consisted of data from non-COVID-19 stroke patients. Data were collected at the time of admission and at discharge. Outcome measures included the functional independence measure (FIM), the Action Research Arm Test (ARAT), the post-COVID-19 functional status (PCFS) scale, the Borg rating of perceived exertion, and the mini-mental state examination (MMSE).

**Results:** Both the post-COVID-19 stroke and non-COVID stroke groups showed significant differences before and after rehabilitation (NIHSS (National Institutes of Health Stroke Scale): *p* = 0.014, 0.000, FIM: *p* = 0.000, 0.000, MMSE: *p* = 0.015, 0.000, ARAT: *p* = 0.000, 0.000, respectively). However, the mean difference in the non-COVID-19 stroke group was higher than that in the post-COVID-19 stroke group, particularly in MMSE, FIM, and NIHSS scores (NIHSS: 2.8 ± 0.4, 0.9 ± 0.04, FIM: 34.8 ± 5.03, 32.95 ± 0.81, MMSE: 5.05 ± 3.5, 0.7 ± 1.17, ARAT: 1 ± 0.062, 1.2 ± 0.47, respectively). It was also found that in the post-COVID-19 stroke group, age had a positive influence on NIHSS (*p* = 0.022) and FIM (*p* = 0.047), and impaired side affected the NIHSS scores (*p* = 0.007). In the non-COVID-19 stroke group, significant correlations were found between the NIHSS and FIM scores (*r* = -0.445, *p* = 0.050) and the NIHSS and ARAT scores (*r* = -0.529, *p* = 0.017).

**Conclusion:** Higher mean differences in the non-COVID-19 stroke group than in the post-COVID-19 group could be due to additional COVID-19 complications in the stroke condition itself. Overall functional gain was observed in both groups due to the effective rehabilitation. Therefore, rehabilitation is critical for functional optimization in such vulnerable populations. There is an urgent need to consider post-pandemic rehabilitation aspects.

## INTRODUCTION

The aftermath of the COVID-19 pandemic has been devastating, resulting in significant mortality and morbidity worldwide. The disabilities and functional limitations experienced by such vulnerable populations have made it challenging for medical and rehabilitation expertise. It is known that stroke survivors and those with post-COVID-19 stroke had different levels of severity in terms of disability and functioning. Rehabilitation outcomes need to be addressed and explored in each of these vulnerable populations to better understand the holistic rehabilitation approach set by the multidisciplinary team. The multidisciplinary team consists of physicians, nurses, occupational therapists, physical therapists, speech and language pathologists, psychologists, social workers, and case managers. People diagnosed with post-COVID-19 stroke may present with additional symptoms and functional limitations than those diagnosed with a stroke alone, according to previous studies. The imperative need for rehabilitation must be the focus. If not addressed early, the disability burden of the patient and their family increases and prevents them from achieving their maximum potential during rehabilitation.

According to recent studies and statistics, the State of Qatar has reported low incidence and mortality rates of stroke at 58 and 9.17 per 100,000 persons per year, respectively.^1^ The five-year average incidence of stroke was 92.04 per 100,000 adults in the Qatari population.^2^ The most common risk factors identified in stroke patients were hypertension (77.9%), diabetes (43.8%), and hypercholesterolemia (28.5%). Stroke occurs at a significantly younger age in Qataris than in the Western population.^2^ Therefore, the burden of stroke is increasing in Qatar, and rehabilitation is of prime importance.

Stroke rehabilitation is a complex process that requires a multidisciplinary team approach. Rehabilitation is a problem-solving process,^3^ and there is ample evidence supporting its effectiveness.^4^ Based on the holistic biopsychosocial model of illness as a framework,^5^ a multidisciplinary team with suitable expertise works on treatment and intervention.^6^ Occupational therapy (OT), a branch of rehabilitation, aims to facilitate task performance by developing either remediating or compensatory strategies to overcome functional deficits that impair the ability to perform daily activities.^7^ The three most commonly chosen OT interventions for stroke patients focus on training in self-care activities, leisure activities, and assistive devices.^8^

Given the global burden of the pandemic and the ultimate need for immediate intervention, it is imperative to understand the rehabilitation process to holistically address and manage these functional impairments. This study will provide a deeper understanding of the rehabilitation and goal-setting processes, as well as an analysis of functional rehabilitation outcomes in terms of functional independence in self-care tasks, upper extremity function, and cognitive function, which are part of the OT assessment and intervention, rather than the overall rehabilitation outcome. This will make it one of the first studies to be conducted in the State of Qatar on such vulnerable populations. Our aim is therefore to compare the functional rehabilitation outcomes of stroke and post-COVID-19 stroke patients following active rehabilitation services from an OT perspective. The other objectives of the study were to study and analyze the functional outcomes of stroke and post-COVID-19 stroke patients, as well as to investigate which factors might influence the functional gain of both groups.

## METHODS

We conducted a mixed-design comparative study to understand and compare the functional outcomes of post-COVID-19 stroke and non-COVID-19 stroke patients. A total of 40 inpatient data were considered for the study. Twenty male inpatients diagnosed with non-COVID-19 stroke who were admitted to the inpatient rehabilitation unit of Qatar Rehabilitation Institute, HMC, Doha, Qatar in 2022 and met the inclusion criteria were prospectively included in the study. The retrospective data of the 20 inpatients diagnosed with post-COVID-19 stroke who were admitted to Qatar Rehabilitation Institute in 2020 were obtained from electronic medical records such as Cerner. Ethical approval was obtained from the Medical Research Center (MRC) before conducting the study (MRC-01-22-015). Subject data were coded using alphanumeric notations. The names of the patients were kept confidential. Only the research team, which included the principal investigator and co-principal investigators, had access to the data. The MRC reviewed the study and raised no ethical objections, following which the study was initiated (MRC-01-22-015).

Sociodemographic variables such as age, marital status, and occupation were obtained from documented records. Other clinical variables that could influence the outcome, such as the extent of infection, the length of stay (LOS) in the ICU or treating hospital, the LOS in quarantine facilities, the date of onset of the infection, and associated conditions such as stroke, polyneuropathy, or myopathy, were also taken into account.

A non-identifiable number was assigned to each patient. The investigators collected the sociodemographic details and also all the study outcome measure scores from the Cerner documentation system at baseline and discharge. They also recorded these data under the assigned non-identifiable participant number. All outcome measures used in this study were both reliable and valid to minimize instrument bias.

Descriptive statistics were used to summarize the baseline characteristics. A paired t-test analysis was used to compare the pre- and post-tests of the study groups at admission and discharge. An independent group t-test or a Wilcoxon signed rank test was used to compare the results of the functional independence measure (FIM), the Action Research Arm Test (ARAT), the mini-mental state examination (MMSE), and Borg rating of perceived exertion (RPE) scores between the two groups. Multiple regression analysis was performed to assess the predictors of functional gain, including age, employment status, impairment side, family history, and LOS. All results are presented with the associated 95% confidence interval. A Shapiro–Wilk test was used to determine the normality of the data. The Shapiro–Wilk test for the post-COVID-19 stroke and non-COVID-19 stroke groups did not show a significant deviation from normality (W(8) = 0.91, *p* = 0.377; W(8) = 0.92, *p* = 0.479).

### Outcome measures

FIM is a primary tool for documenting the patient's functional independence (administered at baseline and every month – seven time points).^9^ The post-COVID-19 functional status (PCFS) scale is a tool to measure functional status over time after COVID-19. It is an ordinal scale that assesses the full range of functional limitations to capture the heterogeneity of post-COVID-19 outcomes. These scales are used to track improvements over time and answer meaningful clinical questions, such as “How will I come out of this corona infection?”, or for research purposes. They can either be self-reported or assessed in a formal standardized interview.^10^ This “PCFS scale” is not currently validated, and its usefulness depends on the local conditions in which it is implemented.

The MMSE questionnaire^11^ consists of 30 questions that are commonly used by doctors and other healthcare professionals to screen for cognitive impairment. The entire test takes about 5–10 minutes. The maximum score for the MMSE is 30. A score of 25 or higher is considered normal. If the score is below 25, the result is usually considered abnormal (indicating possible cognitive impairment). Impairment can be classified as mild (MMSE score between 21 and 24), moderate (MMSE score between 10 and 20), and severe (MMSE score less than 10).

Borg RPE is another outcome measure scale used to determine exercise intensity prescription. It is used to monitor the progress and mode of exercise in cardiac patients and other patient populations undergoing rehabilitation and endurance training. The Borg RPE scale was developed by Gunnar Borg^12^ for rating exertion and breathlessness during physical activity; that is, how difficult the activity is, as evidenced by high heart and respiration rates, profuse perspiration, and muscular exertion. The ARAT is a 19-item observational measure used by physical therapists and other healthcare professionals to assess upper extremity performance (coordination, dexterity, and functioning) during recovery from stroke, brain injury, and multiple sclerosis.^13^ Items comprising the ARAT are categorized into four subscales (grasping, gripping, pinching, and gross movement) and arranged in order of decreasing difficulty, with the most difficult task examined first, followed by the least difficult task.

## RESULTS

**Figure 1.**
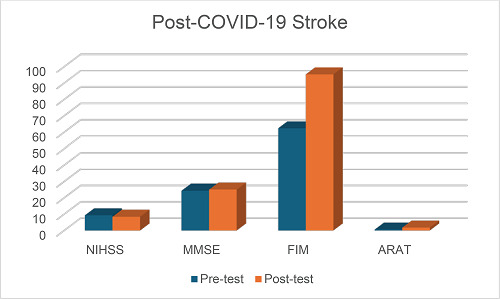
Graphical representation of outcome measures in the post-COVID-19 stroke group.

**Figure 2.**
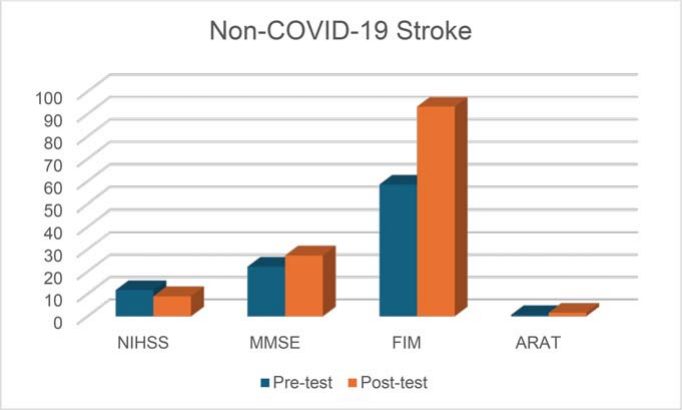
Graphical representation of outcome measures in the non-COVID-19 stroke group.

Demographical data were analyzed in both groups with respect to gender, marital status, employment status, impairment side, and family history (Table 1). Table 2 shows descriptive statistics of both groups. Tables 3 and 4 show the comparison between pre- and post-test outcome (admission–discharge) measures for post-COVID-19 stroke and non-COVID-19 stroke, respectively. Table 5 shows the mean differences between the two groups. Tables 6 and 7 show a comparison of demographic data with functional gain in the post-COVID-19 stroke and non-COVID-19 stroke groups. Figures 1 and 2 show the outcome measures in the post-COVID-19 stroke and non-COVID-19 stroke groups.

## DISCUSSION

The major findings of this study are: (1) significant differences in the outcome measures of both the post-COVID-19 stroke and non-COVID-19 stroke groups (Tables 3 and 4); (2) higher mean differences in the non-COVID-19 stroke group than in the post-COVID-19 stroke group, especially in NIHSS, FIM, and MMSE scores (NIHSS (National Institutes of Health Stroke Scale): 2.8 ± 0.4, 0.9 ± 0.04, FIM:34.8 ± 5.03, 32.95 ± 0.81, MMSE: 5.05 ± 3.5, 0.7 ± 1.17, ARAT: 1 ± 0.062, 1.2 ± 0.47). It was also found that in the post-COVID-19 stroke group, age had a positive influence on NIHSS (*p* = 0.022) and FIM (*p* = 0.047), while impaired side affected NIHSS scores (*p* = 0.007). In the non-COVID-19 stroke group, significant correlations were found between NIHSS and FIM (*r* = -0.445, *p* = 0.050), and NIHSS and ARAT scores (*r* = -0.529, *p* = 0.017).

Very little evidence has been found for OT interventions and their role in predicting and comparing the functional outcomes in stroke and post-COVID-19 stroke patients. Significant physical, cognitive, and psychosocial impairments and disabilities are observed in most stroke survivors,^14^ impeding functional recovery in stroke patients.^15–17^ It is found that nearly 20% of patients remain dependent on self-care after discharge, increasing the burden of care. Furthermore, the prognosis of functional recovery has been observed to be predicted by variables such as severe neurological impairment, activities of daily living (ADL) dependency, cognitive impairment, and stroke recurrence.^18^ Poor functional outcome is strongly correlated with severe neurological impairment,^19–22^ along with a consistent association with post-stroke cognitive decline.^22^ Therefore, adequate rehabilitation is needed to improve cognition, which in turn is associated with improvement in ADL.^23^ Recurrent stroke was significantly associated with ADL dependence compared to first-time stroke.^24,25^ This finding suggests the need to take preventive measures in the early stages of primary care to reduce the impact and severity of stroke. In addition, age, post-stroke depression, and comorbid conditions had a significant impact on ADL dependence.^26^ COVID-19 survivors are more likely to have pre-existing disabling conditions. Prolonged stays in hospital can have physical and psychological effects associated with the severe illness.^27^ One study mentioned that general deconditioning with decreased physical activity was observed during hospitalization.^28^ Critically ill patients may also experience and suffer from functional, social, cognitive, and mental alterations after a prolonged hospital stay,^29,30^ with the quality of life being reduced in a large proportion of patients.^31^ Individuals with delirium are found to have limitations in cognition and areas of basic ADL.^32^

Recently, the emergence of cerebrovascular events leading to acute ischemic strokes associated with COVID-19 has been observed in COVID-19 survivors. Given the increasing reports of such cases, the underlying mechanisms are still uncertain.^33^ In addition, the risk of secondary stroke may be due not only to pathophysiological reasons but also to physical inactivity resulting from isolation and quarantine.^34,35^ Furthermore, stroke survivors who are also infected with COVID-19 have lower modified Rankin scale scores than those without COVID-19 infection.^36^ It has been found that despite significant improvements in most areas, deficits in fall risk, gait speed, and cognition persist at the time of discharge.^37^ Recent studies have suggested rehabilitation strategies and care for stroke patients during the pandemic to prevent secondary stroke and further complications.^29^ Another study explained a stroke care model during the COVID-19 pandemic that aims to preserve patient outcomes while decreasing potential COVID-19 exposure to patients and healthcare providers.^38^ Functional outcomes following a stroke are determined by the type of stroke and motor severity. Advancing age and reduced premorbid function are strongly associated with reduced functional outcomes.^39,40^ According to the literature, age and impaired side were also found to be a significant predictor of functional gain and outcomes in post-COVID-19 stroke patients.^41^ Therefore, it is essential to study the functional outcomes of both vulnerable groups from the OT perspective to better understand rehabilitation needs and processes.

It is important to observe positive changes in the motor and functional status of patients with rehabilitation potential to estimate prognosis and rehabilitation goals.^42^ This can enable clinicians to assess and expect outcomes within the rehabilitation program and plan their goals accordingly. When considering both groups, NIHSS, cognition, FIM, and ARAT showed significant changes in pre- and post-test scores (*p* < 0.05) (Tables 3 and 4). A significant difference was that patients in both groups showed improvements during their rehabilitation period. A study by Paolucci et al.^43^ also mentioned that rapid rehabilitation is essential to enable adequate functional recovery. This supports the fact that the interdisciplinary team has brought about positive changes in function through its holistic approach.

The significant changes in NIHSS and cognition were more pronounced in the non-COVID-19 stroke group (*p* = 0.000) than in the post-COVID-19 stroke group (*p* = 0.014; *p* = 0.015, respectively). While both groups showed equal significant changes in FIM and ARAT (*p* = 0.000). The slightly better scores in the non-COVID-19 stroke group compared with the post-COVID-19 stroke group with respect to NIHSS and cognition could be due to complications after COVID-19 sequelae in the latter, which could have slowed recovery. This can be supported by studies reporting declines in cognitive function and working memory observed during the pandemic.^44^ Cognitive decline in hospitalized patients could be due to differences in settings,^41^ which has been shown to have a significant impact on functional outcomes.^45^ Another study also mentioned that stroke patients who had COVID-19 had an increased potential to develop long-term cognitive deficits and progress to dementia.^46^

Stroke severity and outcomes assessed by NIHSS scores are good predictors of the functional recovery and prognosis of stroke patients. Studies have shown that mean NIHSS scores were higher in stroke patients with COVID-19 infection than in patients without COVID-19. This was because the functional outcomes of patients with COVID-19 were poorer when compared with those without COVID-19 due to the clinical characteristics and severity of disability caused by COVID-19.^31^ This was demonstrated in the present study by a significant difference in the NIHSS scores in both groups. Thus, the results showed that NIHSS significantly influenced the functional recovery of both groups.

The mean differences between the two groups showed that there were significant differences in the MMSE (P < 0.0001) and ARAT (*p* = 0.044) scores. The two groups did not differ in FIM, proving that rehabilitation was effective in both groups (*p* = 0.112). Cognition as measured by the MMSE tool was affected in stroke patients following the COVID-19 pandemic, as were the usual trends of cognitive decline. This can be demonstrated in post-COVID-19 stroke patients who suffer from varying degrees of cognitive impairment, which affects the overall quality of life due to the COVID-19 infection and leads to cognitive limitations due to its pathogenesis and underlying mechanisms.^47^ The other aspect to investigate is the significant mean differences in arm and hand functions as measured by ARAT. Due to the intensity of rehabilitation, significant improvements in arm and hand function were observed, which facilitated functional recovery. The differences in functional gain and scores on the outcome measures were more significant compared to the post-COVID-19 stroke group, which could be due to reasons such as increased severity, prolonged hospitalization due to the complexity of COVID-19 infection with stroke, and fewer with good recovery status.^48^ This may explain why there were better functional outcomes in the non-COVID-19 stroke group than in the post-COVID-19 stroke group.

In the post-COVID-19 stroke group, the effect of age and impairment side was shown to positively influence the NIHSS (*p* = 0.022, *p* = 0.007, respectively) and FIM (*p* = 0.047) scores. One study found that acute ischemic stroke associated with COVID-19 has received considerable attention on the effect of age on such factors. It showed that COVID-19 ischemic strokes were much more likely to affect younger patients than pre-pandemic controls. It has also been studied that poorer functional outcomes and mortality rates are associated with age.^49^

In the non-COVID-19 stroke group, significant correlations were found between NIHSS and FIM scores (*r* = -0.445, *p* = 0.050) and NIHSS and ARAT scores (*r* = -0.529, *p* = 0.017). One study determined whether impairment due to stroke, as measured by neurological examination scores (NIHSS), correlated with disability as measured by the FIM. A highly significant correlation was found between NIHSS and FIM (*p* < 0.0001), confirming that NIHSS scores are a good predictor of stroke disability in terms of FIM and functional gain.^50^ One study also found that there was a significant association between NIHSS and upper extremity impairment and stroke severity.^51^ This may explain why there was a significant correlation between NIHSS and ARAT scores for assessing arm function in stroke patients in the present study.

There are strengths to our study. This study is one of the few that has recorded the comparison of functional outcomes between two vulnerable groups. It is also noteworthy that the rehabilitation of these vulnerable populations was successful and met the goals set by the multidisciplinary team. The study also has some limitations. These include a small sample size and therefore cannot be generalized to a larger population, and therefore needs to be investigated. In addition, the heterogeneity of the groups could be another limitation. The sample size included both ischemic and hemorrhagic strokes, which could lead to differences in overall results due to varying clinical characteristics and outcomes.

## CONCLUSION

The impact of COVID-19 infection on stroke is noteworthy, as shown by the results of this study. The pandemic and infection were found to have a negative impact on functional outcomes and have a debilitating effect on the overall quality of life and well-being. Immediate and timely intervention is needed to effectively rehabilitate these vulnerable populations. Positive functional gain was noted in both groups, indicating the effectiveness of rehabilitation. Further studies are needed to explore such functionalities and demonstrate the need for rehabilitation.

### List of abbreviations

[Table tbl8]

### Competing interests

The authors declare that there is no conflict of interest.

### Data availability

The statistical analysis and data used to support the findings of this study are included in the article. The other raw data used can be provided upon request.

### Authors’ contributions

All the authors contributed significantly to the conception and design of the study. All the authors read and approved the final manuscript.

### Acknowledgments

The authors would like to thank the entire therapy team and the hospital for their continued support in completing the study. A special mention and gratitude goes to our patients, without whom this study would not be possible.

## Figures and Tables

**Table 1:** Demographic details.

	Post-COVID-19 stroke (*n*)	Non-COVID-19 stroke (*n*)	Post-COVID-19 stroke (%)	Non-COVID-19 stroke (%)

Gender (male)	20	20	100	100

Marital status (married)	20	20	100	100

Employment status				

(a) Retired	5	2	25	10

(b) Working	15	18	75	90

Impairment side				

(a) Bilateral	2		10	

(b) Left	13	12	65	60

(c) Right	5	8	25	40

Family history				

(a) With	17	12	85	60

(b) Without	3	8	15	40

Type of stroke				

(a) Ischemic	16	7	80	35

(b) Hemorrhagic	4	13	20	65


**Table 2:** Descriptive statistics.

Post-COVID-19 stroke	Mean	SD

Age	55.70	8.820

LOS (in days)	62.45	37.618

Admission (NIHSS score)	9.30	2.452

Discharge (NIHSS score)	8.40	2.415

Cognition at the time of admission (MMSE)	24.35	1.565

Cognition at the time of discharge (MMSE)	25.05	1.234

Admission (FIM)	62.35	21.847

Discharge (FIM)	95.30	21.032

Non-COVID-19 stroke		

Age (years)	54.20	11.228

LOS (in days)	51.75	13.498

Admission (NIHSS score)	11.75	2.511

Discharge (NIHSS score)	8.95	2.188

Cognition at the time of admission (MMSE)	22.20	5.917

Cognition at the time of discharge (MMSE)	27.25	2.468

Admission (FIM)	58.50	17.074

Discharge (FIM)	93.25	12.703


**Table 3:** Comparison of outcomes in the post-COVID-19 stroke group.

	Pre-test scores, mean (SD)	Post-test scores, mean (SD)	Diff. scores	*t*	*p*

NIHSS score	9.30 (2.45)	8.40 (2.41)	1.59	2.714	0.014

Cognition	24.35 (1.56)	25.05 (1.23)	-0.150	-2.666	0.015

FIM	62.35 (21.84)	95.30 (21.03)	-28.785	-16.557	0.000

PCFS	3.55 (0.82)	2.05 (1.19)	2.015	6.097	0.045

ARAT	0.55 (0.60)	1.75 (1.07)	-0.841	-6.990	0.000


**Table 4:** Comparison of outcomes in the non-COVID-19 stroke group.

	Pre-test scores, mean (SD)	Post-test scores, mean (SD)	Diff. scores	*t*	*p*

NIHSS score	11.75 (2.51)	8.95 (2.18)	3.45	8.949	0.000

Cognition	22.20 (5.91)	27.25 (2.46)	-3.056	-5.300	0.000

FIM	58.50 (17.07)	93.25 (12.70)	-27.528	-10.072	0.000

ARAT	0.60 (0.82)	1.60 (0.88)	-0.696	-6.892	0.000


**Table 5:** Between-group analysis.

	Non-COVID-19 stroke, mean ± SD	Post-COVID-19 stroke, mean ± SD	*t*	*p*

FIM	34.8 ± 5.03	32.95 ± 0.81	-1.624	0.112

MMSE	5.05 ± 3.5	0.7 ± 1.17	-5.271	

ARAT	1 ± 0.062	1.2 ± 0.47	1.887	0.044


**Table 6:** Comparison of demographics with functional gain in the post-COVID-19 stroke group.

	Demographics	Coefficient	*t*	*p*

NIHSS	Age	-0.769	-2.584	0.022*

	Impairment side	0.573	3.185	0.007*

FIM	Age	-0.764	-2.173	0.047*


*Statistically significant at *p* < 0.05.

**Table 7:** Correlations of demographics with functional gain in the non-COVID-19 stroke group.

	Demographics	Pearson’s correlation	*p*

NIHSS	FIM	-0.445	0.050*

	ARAT	-0.529	0.017*


*Statistically significant at *p* < 0.05.

**Table tbl8:** 

ADL	Activities of Daily Living

ARAT	Action Research Arm Test

FIM	Functional Independence Measure

MMSE	Mini-Mental State Examination

NIHSS	National Institutes of Health Stroke Scale

PCFS	Post-COVID-19 Functional Status

RPE	Rate of Perceived Exertion

